# A Model to Detect Significant Prostate Cancer Integrating Urinary Peptide and Extracellular Vesicle RNA Data

**DOI:** 10.3390/cancers14081995

**Published:** 2022-04-14

**Authors:** Shea P. O’Connell, Maria Frantzi, Agnieszka Latosinska, Martyn Webb, William Mullen, Martin Pejchinovski, Mark Salji, Harald Mischak, Colin S. Cooper, Jeremy Clark, Daniel S. Brewer, on behalf of The Movember GAP1 Urine Biomarker Consortium

**Affiliations:** 1 Norwich Medical School, University of East Anglia, Norwich Research Park, Norwich NR4 7TJ, UK; sheaconnell@gmail.com (S.P.O.); m.webb@uea.ac.uk (M.W.); colin.cooper@uea.ac.uk (C.S.C.); jeremy.clark@uea.ac.uk (J.C.); 2 Department of Biomarker Research, Mosaiques Diagnostics GmbH, 30659 Hannover, Germany; frantzi@mosaiques-diagnostics.com (M.F.); latosinska@mosaiques-diagnostics.com (A.L.); pejchinovski@mosaiques-diagnostics.com (M.P.); mischak@mosaiques.de (H.M.); 3 Institute of Cardiovascular and Medical Sciences, University of Glasgow, Glasgow G12 8TA, UK; william.mullen@glasgow.ac.uk; 4 Institute of Cancer Sciences, University of Glasgow, Glasgow G61 1BD, UK; mark.salji@glasgow.ac.uk; 5 The Earlham Institute, Norwich Research Park, Norwich NR4 7UZ, UK

**Keywords:** extracellular vesicles, mass spectrometry, prostate cancer, urinary biomarkers, RNA

## Abstract

**Simple Summary:**

Prostate cancer is one of the leading causes of cancer-related death in men in the world, but a large proportion of men that are diagnosed with prostate cancer do not have a form of the disease that will cause them long term harm. Therefore, there is a need to accurately predict the aggressiveness of the disease without taking an invasive biopsy. In this study, we develop a test that can predict whether a patient has prostate cancer and how aggressive that cancer is. This test combines clinical measurements, levels of four genes collected from a fraction of the urine, and levels of six peptides found in urine. We found that this test, deemed ‘ExoSpec’, has the potential to improve the pathway for men with a clinical suspicion of prostate cancer and could reduce the requirement for biopsies by 30%.

**Abstract:**

There is a clinical need to improve assessment of biopsy-naïve patients for the presence of clinically significant prostate cancer (PCa). In this study, we investigated whether the robust integration of expression data from urinary extracellular vesicle RNA (EV-RNA) with urine proteomic metabolites can accurately predict PCa biopsy outcome. Urine samples collected within the Movember GAP1 Urine Biomarker study (*n* = 192) were analysed by both mass spectrometry-based urine-proteomics and NanoString gene-expression analysis (167 gene-probes). Cross-validated LASSO penalised regression and Random Forests identified a combination of clinical and urinary biomarkers for predictive modelling of significant disease (Gleason Score (Gs) ≥ 3 + 4). Four predictive models were developed: ‘MassSpec’ (CE-MS proteomics), ‘EV-RNA’, and ‘SoC’ (standard of care) clinical data models, alongside a fully integrated omics-model, deemed ‘ExoSpec’. ExoSpec (incorporating four gene transcripts, six peptides, and two clinical variables) is the best model for predicting Gs ≥ 3 + 4 at initial biopsy (AUC = 0.83, 95% CI: 0.77–0.88) and is superior to a standard of care (SoC) model utilising clinical data alone (AUC = 0.71, *p* < 0.001, 1000 resamples). As the ExoSpec Risk Score increases, the likelihood of higher-grade PCa on biopsy is significantly greater (OR = 2.8, 95% CI: 2.1–3.7). The decision curve analyses reveals that ExoSpec provides a net benefit over SoC and could reduce unnecessary biopsies by 30%.

## 1. Introduction

Prostate cancer (PCa) ranks as the second most commonly diagnosed cancer among men [1]. Although this malignancy is diagnosed in about one in eight men, 78% survive prostate cancer for 10 or more years [2]. PCa is a heterogeneous disease with many men presenting with low-risk indolent disease that is unlikely to progress, while others have aggressive clinically significant life-threatening disease requiring treatment intervention.

Clinical tests to predict the presence and aggressiveness of PCa on biopsy include serum PSA, digital rectal examination (DRE), and more recently MRI. However, PSA lacks specificity, with only ~40% of all patients with an elevated PSA (≥4 ng/mL) being positively confirmed with PCa on biopsy [3]. DRE has been reported to be subjective and lumps felt by DRE can disappear [4]. MRI has high sensitivity for significant disease but also a high false positive rate of ~50% [5]. Accurate discrimination between slow growing and aggressive PCa remains a major challenge. This is reflected in over-treatment of patients with indolent disease and under-treatment of those with lethal disease.

The prostate is a secretory organ. Prostatic secretions flow into the urethra and are flushed out on urination. Prostate cancer cells, extracellular vesicles (EVs), and molecules are transported in the prostatic secretions and can be detected in urine, which has been shown to be a non-invasive source of biomarkers for prostate cancer [6]. Single- or few-biomarker panels such as PCA3 [7], SelectMDx [8], and ExoDx Prostate (IntelliScore) [9] tests have been published. However, they are in various stages of clinical validation and none are currently implemented in the UK healthcare system [10]. In 2011 we initiated the collection of a large set of samples (the Movember GAP1 Urine biomarker cohort) which were analysed by a range of methods with the aim of determining the best means of analysing urine for diagnostic and prognostic PCa biomarkers. Analyses included ELISA, mass spectrometry, RT-PCR, DNA-methylation patterns, and RNA expression data from urine cell pellet and urine extracellular vesicles. Four risk classifiers for significant PCa have so far been developed and published: (i) ‘PUR’ (Prostate Urine Risk) signatures using expression data for 167 gene-probes in urine-derived Extracellular vesicle RNA (EV-RNA), which provided additional prognostic information for men on Active Surveillance (AS) [11], (ii) ‘ExoMeth’ integrating cell pellet methylation data with urine EV-RNA data in a subset of samples [12], (iii) ‘ExoGrail’ which integrates whole urine EN2 protein ELISA data with urine EV data in a subset of samples [13], and (iv) Applying machine learning algorithms to urine proteomic data collected by mass spectrometry to generate proteomics patterns which could identify advanced cancers (Gs ≥ 3 + 4) [14]. All four risk models showed promising results with AUCs for detecting significant cancer of 0.77, 0.84, 0.89, and 0.8, respectively.

Here, we aim to investigate whether robust integration of urine EV-RNA data with CE-MS proteomic features and clinical data in multivariable models can improve the accuracy of predicting clinically significant PCa found on biopsy. The results of this study connect with our other Movember cohort studies [11,12,13,15,16] which together provide valuable information on which combination of urine markers have the potential to improve the accuracy of predicting clinically significant PCa in biopsy naïve men.

## 2. Materials and Methods

### 2.1. Patient Population and Characteristics

Urine samples were collected for the Movember GAP1 Urine Biomarker Cohort between 2009 and 2015. Consenting men attending urology clinics at multiple sites provided first-catch urine samples, collected post-DRE and pre-biopsy (fully described by Connell et al. [11]). Inclusion criteria for model development were that urine samples had been analysed by both extracellular vesicle RNA analysis (NanoString) and mass spectrometry analysis of excreted urinary peptides. Exclusion criteria: Men who had had a prostate biopsy or trans-urethral resection of the prostate up to 6 weeks previously and men with metastatic cancer (positive bone scan or PSA > 100 ng/mL).

All samples analysed in the ExoSpec cohort were collected from the Norfolk and Norwich University Hospital (NNUH, Norwich, UK; Table 1). Sample collections and processing were ethically approved by the East of England REC. D’Amico classification used Gleason Score (Gs) and PSA criteria as per D’Amico et al. [17]. All biopsies were TRUS guided. Where subsequent biopsies were taken the results from the closest biopsy to initial urine sample collection were used. The ‘No Cancer’ (NC) patient group (*n* = 59) were a combination of men with raised PSA and a negative biopsy for cancer (*n* = 36) and men with no evidence of cancer (*n* = 23) who had a PSA normal for their age [18], a normal DRE, and for whom there was no suspicion of prostate cancer and as such had not been biopsied.

### 2.2. Sample Collection and Processing

Urine samples were collected, processed, and the extracellular vesicle RNA was extracted according to the Movember GAP1 standard operating procedure, as previously described by Connell et al. [11].

### 2.3. NanoString Analysis

NanoString analysis of extracellular vesicle RNA (EV-RNA) was performed as described in Connell et al. [11], with the modification that NanoString data were normalised according to NanoString guidelines using NanoString internal positive controls and log_2_ transformed. The NanoString data presented here are a subset (*n* = 192) of the data previously reported by Connell et al. [11] for which mass spectrometric analysis had also been performed. See Table S1 for NanoString probe sequences.

### 2.4. Mass Spectrometry Analysis

Capillary electrophoresis–mass spectrometry (CE-MS) analyses were performed on whole urine samples (*n* = 192) stored at −80 °C using the previously established protocols for sample preparation and data acquisition [19]. CE-MS analysis and data processing were performed according to the ISO13485 quality standard [20]. In short, samples (700 µL) were mixed 1:1 with alkaline buffer (2 M urea, 10 mM NH_4_OH, and 0.02% SDS (pH 10.5)), filtered with Centrisart ultracentrifugation filters (Sartorius, Göttingen, Germany) to retain proteins/polypeptides below 20 kDa. To remove urea, electrolytes, and salts and to decrease matrix effects, the samples were ultra-filtrated using Centrisart ultracentrifugation filter devices (20 kDa MWCO; Sartorius, Goettingen, Germany) at 3000 rcf until 1.1 mL of filtrate was obtained. Later, the volume of 1.1 mL of the filtrate was applied on PD-10 columns (GE Healthcare, Munich, Germany) equilibrated with 0.01% NH_4_OH in high-performance liquid chromatography (HPLC)-grade H_2_O (Carl Roth GmbH, Karlsruhe, Germany). After rinsing the column with 1.9 mL of 0.01% NH_4_OH in H_2_O, 2 mL of HPLC-grade H_2_O was applied, and the resulting eluate was collected. The eluate was lyophilized and resuspended in HPLC-grade H_2_O shortly before analysis, as previously described [21]. The analysis was performed using a P/ACE MDQ capillary electrophoresis system (Beckman Coulter, Fullerton, CA, USA) coupled with a Micro-TOF MS (BrukerDaltonic, Bremen, Germany) supplemented with 0.94% formic acid (Merck KGaA, Darmstadt, Germany sourced from Sigma-Aldrich) as running buffer. In addition, the electrospray ionization interface from Agilent Technologies (Palo Alto, CA, USA) was set to a potential of −4.0 to −4.5 kV. Spectra were recorded over an m/z range of 350–3000 and accumulated every 3 s [21].

### 2.5. Peptidomic Data Processing

Mass spectral ion peaks representing identical molecules at different charge states were de-convoluted into single masses using MosaiquesVisu software [20,22]. Normalisation of the CE-MS data was based on twenty-nine collagen fragments that are generally not affected by disease and serve as internal standards [23]. A mass spectrometric peak list for each peptide was defined by its molecular mass (kDa), normalized migration time (min), and normalized signal intensity (AU) [22]. Normalization of the CE-MS data was based on 29 internal collagen fragments found to be stable over disease/health state that served as internal standards [23]. All detected peptide data were deposited, matched, and annotated in a Microsoft SQL database and used as input in the presented study [24]. Polypeptides obtained from different samples were considered identical when mass deviation was 50 ppm for peptides of 800 kDa and 100 ppm for peptides with a maximum mass of 20 kDa. Due to analyte diffusion effect, CE peak widths increase with CE migration time. For data clustering this effect is considered by linearly increasing cluster widths over the entire electropherogram (19 min to 45 min) from 2 to 5%. Transformation of the data (log-transformation) was performed before performing the statistical analysis, as previously described [25]. These data have not been described before and are unique to this study.

### 2.6. Peptide Sequence Assignment

Matching of the amino acid sequences with ion peaks obtained by CE-MS was based on mass correlation between CE-MS and liquid chromatography-tandem mass spectrometry analysis (LC-MS/MS). Further validation of the obtained peptide identifications was based on the assessment of the peptide charge at the working pH of 2.2 and the CE-migration time results [20]. The amino acid sequences were obtained by performing MS/MS analysis using either a PACE CE or a Dionex Ultimate 3000 RSLS nanoflow system (Dionex, Camberly, UK) coupled to an Orbitrap Velos instrument (Thermo Fisher Scientific Inc., Boston, USA), as previously described [26]. The mass spectrometer was operated in MS/MS mode scanning from 350 to 1500 amu. The fragmentation method was HCD at 40% collision energy. For CE, the top five multiple charged ions were selected for each scan for the MS/MS analysis whereas for LC, the top 20 multiple charged ions were selected for each scan for MS/MS. The detection limit for the LC- or CE-MS/MS analysis using the Orbitrap Velos mass spectrometer, with 60,000 resolution for MS1 and with 7500 resolution for MS2, was in the range of 0.05–0.2 fmol [27]. Proteins and peptides were searched against Uniprot human non-redundant database (fasta file version from 20 June 2019) using Proteome Discoverer 1.4 (activation type: HCD; precursor mass tolerance: 5 ppm; fragment mass tolerance: 0.05 Da) without enzyme specificity. No fixed modification was selected. Oxidation of proline and methionine (indicated with ‘p’ and ‘m’) as well as deamidation (indicated with ‘q’) were set as variable modifications. High confidence peptides with Xcorr ≥ 1.9 and rank 1 were accepted as most valid for identification of the peptide markers (Pejchinovski et al. 2015). Sequences that were not successfully matched to correct peptide markers under these criteria were not reported (indicated with ‘-‘).

### 2.7. Statistical and Data Analysis

Peptide data were filtered a priori by only retaining peptides quantified at any level in at least 30% of either cancer or non-cancer samples. All analyses, model construction, and data preparation were undertaken in R version 3.5.3 [28], and unless otherwise stated, utilised base R and default parameters. All data and the code and scripts required to reproduce these analyses can be found at https://github.com/UEA-Cancer-Genetics-Lab/ExoSpec (accessed on 13 March 2022).

#### 2.7.1. Feature Selection Using LASSO (Least Absolute Shrinkage and Selection Operator)

EV-RNA, CE-MS, and clinical markers were interrogated for useful information. Following filtering, a dataset comprised a total of 814 possible variables for predictive modelling including EV-RNA (*n* = 167), peptides (*n* = 643), and clinical variables (*n* = 4) was derived. Subsequently, feature selection was performed as a key task to minimise the potential for model overfit and increase the robustness of any trained models. Variables robustly associated with Gleason Score were identified by means of a 20-fold cross-validated LASSO (L_1_-penalised) generalised linear model, fit using the *glmnet* package [29]. Only features whose coefficients were not decreased to zero by LASSO were considered further and were positively selected as input to Random Forest-based comparator models.

#### 2.7.2. Model Construction

All models were trained via the Random Forest algorithm [30], using the *randomForest* package [31] with default parameters except for: (a) resampling without replacement and (b) 401 decision trees being grown per model. Risk scores, as generated by the trained models, are presented as the out-of-bag predictions: the aggregated outputs from decision trees within the forest where the sample in question has not been included within the resampled dataset [30]. Both cross-validation folds and bootstrap resamples were identical for feature selection and model training, respectively, for all models and by applying the same random seed. Models were trained on a modified continuous outcome (range: 0–1) based on the dominant Gleason pattern: where no evidence of cancer was set to 0, Gleason scores 3 + 3 & 3 + 4 to 0.5, and Gleason scores ≥ 4 + 3 to 1. Using Gleason score as a continuous variable better reflects the reality that two patients with the same TRUS-biopsy Gleason score will not share the exact same proportions of tumour pattern or overall disease burden within their prostate. Following this categorisation, the score is treated as a continuous variable by the Random Forest algorithm described above. When determining the predictive ability and clinical utility of the models, the original non-continuous Gleason score is used.

#### 2.7.3. Comparator Models

To evaluate potential clinical utility, additional models were trained as comparators using subsets of the available variables across the patient population: a clinical standard of care (‘SoC’) model was trained by incorporating age, PSA, T-staging, and clinician DRE result; a model using only the pre-filtered CE-MS derived peptides (‘MassSpec’, *n* = 643); and a model only using NanoString gene-probe information (‘Exo-RNA’, *n* = 167). The fully integrated ‘ExoSpec’ model was trained by incorporating information from all the above variables (*n* = 814). Each set of variables were independently selected for generating comparator models via the cross-validated LASSO feature selection process as described above to select the optimal subset of variables possible for each predictive model.

#### 2.7.4. Statistical Evaluation of Model Predictivity

Metrics for Area Under the Receiver-Operating Characteristic curve (AUC) were produced using the *pROC* package [32], with confidence intervals calculated via 1000 stratified bootstrap resamples. Density plots of model risk scores and all other plots were created using the *ggplot2* package. Cumming estimation plots and calculations were produced using the *dabestr* package [33] and 1000 bootstrap resamples were used to visualise robust effect size estimates of model predictions. Decision curve analysis (DCA) [34] examined the potential net benefit of using the developed risk-signatures in the clinic. Standardised net benefit (sNB) was calculated with the *rmda* package [35] and presented throughout our decision curve analyses as it is a more directly interpretable metric compared to net benefit [36]. To ensure DCA was representative of a more general population, the prevalence of Gleason scores within the ExoSpec cohort were adjusted via bootstrap resampling to match those observed in a population of more than 219,000 men within the control arm of the Cluster Randomised Trial of PSA Testing for Prostate Cancer (CAP) Trial [37], as described in Connell et al. [11]. Briefly, the biopsied men within this CAP cohort were 23.6% GS 6, 8.7% GS 7, and 7.1% GS ≥ 8, with 60.6% of biopsies showing no evidence of cancer. These ratios were used to perform stratified bootstrap sampling with replacement of the Movember cohort to produce a new dataset of 197 samples with risk scores from each comparator model. sNB was then calculated for this resampled dataset, and the process repeated for a total of 1000 resamples with replacement. The mean sNB for each risk score and the treat-all options over all iterations were used to produce the presented figures to account for variance in resampling. Net reduction in biopsies was calculated relative to the clinical Standard of Care model, as it is the best decision model we could produce with the clinical data within this cohort as opposed to defaulting to undertaking biopsy in all patients with a PSA ≥ 4 ng/mL. With this considered, biopsy reduction was calculated as:

(1) $$Biopsy_{NetReduction}=\left(NB_{Model}-NB_{SoC}\right)\times \frac{1-Threshold}{Threshold},$$

where the decision threshold (*Threshold*) is determined by accepted patient/clinician risk. For example, a clinician may accept up to a 25% perceived risk of significant cancer before recommending biopsy to a patient, equating to a *Threshold* of 0.25.

## 3. Results

### 3.1. The Development Cohort

The development cohort consisted of paired extracellular vesicle RNA (EV-RNA) and mass spectrometry peptide-metabolite datasets derived from urine collected from 192 patients during Movember GAP1 Urine biomarker study (Table 1).

### 3.2. Feature Selection and Model Development

Using LASSO regression models based on 20-fold cross-validation, feature selection was performed for four datasets: only clinically available parameters, the EV-RNA dataset, the mass spec dataset, and the integrated dataset combining all three types of data (Table 2). LASSO regression will select those features (EV-RNA probes, peptides, or clinical variables) that are useful in predicting risk category, discarding the redundant or useless features. Of the clinical data (serum PSA, age at sample collection, DRE impression, and urine volume collected) only age and PSA were selected as significant predictors of biopsy outcome, both increased in prostate cancer (PCa) patients. Following filtering of the mass spec data, 643 peptides were inputted into the feature selection, of which 14 were found to have significant utility in predicting PCa biopsy outcome (Table 2, Table S1). Nine peptides were detected in higher levels in urine from PCa patients; these included fragments of matrix metalloproteinase-2 (MMP2, 4.8× higher), three peptide fragments of fibrinogen alpha chain (FGA, 3.2–5.6×), and Histone H1.4 (HIST1H1E, 7.1×). Five peptides were detected in decreased abundance: glutamate dehydrogenase 1 (GLUD1, 2-fold decrease in PCa men) and four collagen peptides. Three fragments of collagen 1 alpha 1 (COL1A1) had significant utility; one was upregulated in PCa samples (6.4×) and two were downregulated (0.6–0.7×).

For the EV-RNA dataset, six transcripts were selected (Table 2, Table S1). Four were identified in increased urinary abundance in men with PCa (4.8×), including *ERG* (*ETS Transcription Factor,* 4.8×) and *PCA3 (prostate cancer antigen 3*, 4.2×). Two genes (*SNORA20* (small nucleolar RNA) and *SERPINB5* (serine protease inhibitor) were at higher levels in men with no evidence of cancer).

The above selected features were subsequently used to train four Random Forest based comparator models: (1) a standard of care (‘SoC’) model using only clinically available information, (2) a ‘MassSpec’ model using only peptide mass spectrometry data (fourteen predictive peptides), (3) an ‘ExoRNA’ model using only EV-RNA information (six gene-probes), and (4) a multi-omics integrated model combining all data deemed ‘ExoSpec’.

### 3.3. Comparative Assessment of the Four Predictive Models

The three models using only a single dataset performed reasonably well with area under the receiver operator curves (AUCs) for detection of any cancer ranging from 0.76 to 0.84 and AUCs for detection of Gleason score (Gs) ≥ 3 + 4 of ≥0.69–0.75 (Table 3). ExoSpec AUC values were superior to all single-data models in predicting presence of any cancer, Gs ≥ 3 + 4, and Gs ≥ 4 + 3 (ExoSpec AUCs 0.91, 0.83, 0.82, respectively, all *p* < 0.001, bootstrap test, 1000 resamples, Table 3).

**Figure 1:**
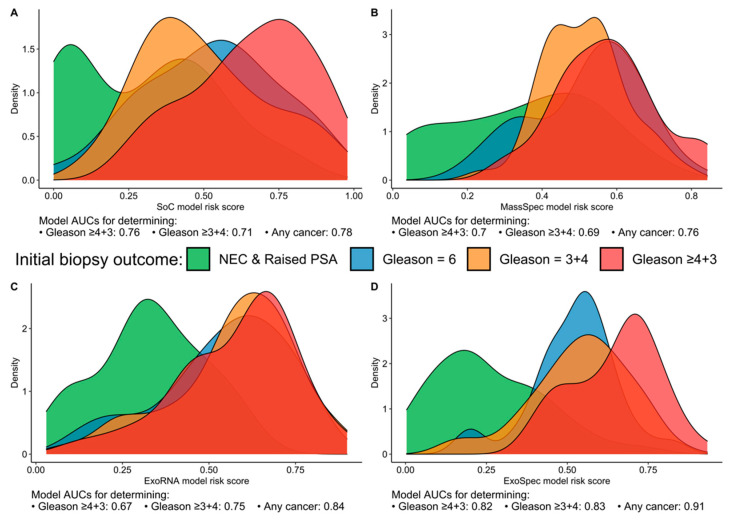
Risk score distributions generated by the four models divided by biopsy outcome data. (**A**) SoC—model derived from clinical variables; (**B**) MassSpec—model built using mass spec peptide data; (**C**) ExoRNA—model derived from EV-RNA information; and (**D**) ExoSpec—model using the integrated clinical, peptide and EV-RNA data. Distributions are coloured according to biopsy outcome: green—No Cancer (No Evidence for Cancer (NEC) and Raised PSA negative biopsy samples), blue—Gleason score (Gs) 3 + 3, orange—Gs 3 + 4, red—Gs ≥ 4 + 3).

When we examined the distribution of patients for each model’s risk score, we found that the SoC model was able to discriminate the ‘No Cancer’ from the highest risk patients (Gs ≥ 4 + 3) with good accuracy but could not separate Gs3 + 3 from clinically significant Gs3 + 4 disease, with the latter possessing a lower mean SoC risk score (Figure 1A). Neither the MassSpec nor the ExoRNA comparator models could effectively differentiate between the three cancer groups (Gs3 + 3, Gs3 + 4, Gs ≥ 4 + 3); however, ExoRNA was much better at separating PCa and No Cancer (NC) samples (AUC 0.84, Figure 1B,C). The multimodal ExoSpec model displayed clear improvements in separating the NC from the other cancer groups (Figure 1D) and improved discernment of men with Gs ≥ 4 + 3 from men with majority Gleason 3 cancers.

**Figure 2:**
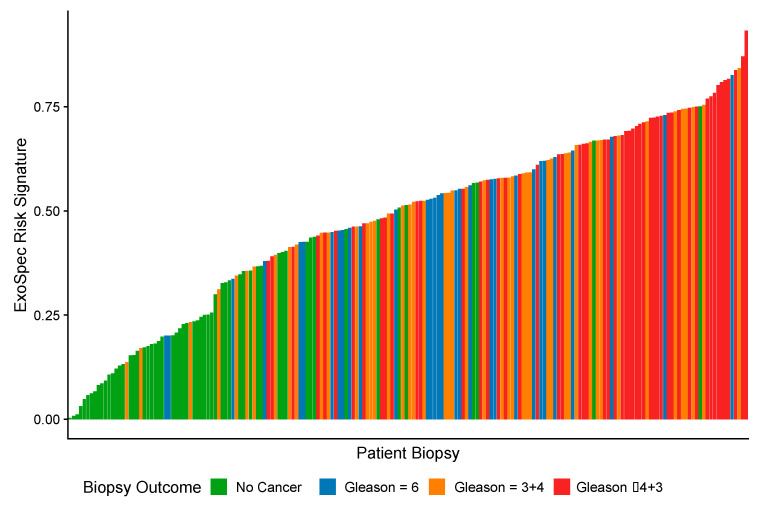
A waterfall plot showing how ExoSpec risk score varies with biopsy outcome (increasing Gleason score (Gs) is associated with more aggressive disease). The height of each coloured bar is the predicted risk score given by the ExoSpec model for an individual biopsy. The colour of the bar represents the biopsy outcome: green—No Cancer; blue—Gs 3 + 3; orange—Gs 3 + 4, red—Gs ≥ 4 + 3.

As ExoSpec Risk Score (range 0–1) increased, the likelihood of high-grade disease being detected on biopsy was significantly greater (proportional odds ratio = 2.26 per 0.1 ExoSpec risk score increase, 95% CI: 1.91–2.71; ordinal logistic regression; displayed as a waterfall plot in Figure 2).

**Figure 3:**
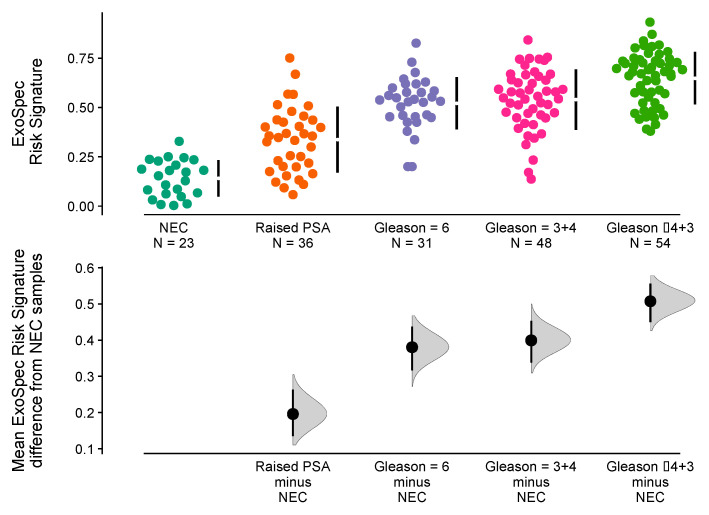
Relationship between ExoSpec risk signature score and biopsy status using Cumming estimation plots. In the top panel, each point displays the ExoSpec risk score for a sample stratified by biopsy status across the x-axis. Each sample point is coloured according to their biopsy Gleason score or ‘No Cancer’ status: NEC—No evidence of cancer, Raised PSA—Raised PSA with negative biopsy. Mean and standard deviation ExoSpec risk signature score distributions for each group are shown by a gapped vertical line. The bottom panel shows mean differences in ExoSpec signatures relative to NEC patient samples. Calculated from bias-corrected and accelerate bootstrap resampling (1000 resamples with replacement), sample density distributions are presented with a point estimate and vertical bar to show mean difference and 95% confidence intervals, respectively.

The ‘No Cancer’ samples (*n* = 59) were separated into two subgroups namely (i) No Evidence of Cancer (‘NEC’, PSA normal for age, no biopsy, *n* = 23) and (ii) Raised PSA Negative Biopsy patients (*n* = 26). Mean ExoSpec scores for each of the clinical groups were calculated after 1000 bias-corrected and accelerated bootstrap resamples (Figure 3). Notably, the Raised PSA Negative Biopsy patients had a higher ExoSpec risk score than NEC (mean difference = 0.2 (95% CI: 0.13–0.26)) and exhibited a wider ExoSpec score distribution than other clinical categories, suggesting these patients may not form a homogenous molecular or biological group. Mean ExoSpec score differences between NEC and the three cancer subgroups were as follows: Gs3 + 3 = 0.38 (95% CI: 0.32–0.44), Gs3 + 4 = 0.4 (95% CI: 0.34–0.45), and Gs ≥ 4 + 3 = 0.51 (95% CI: 0.45–0.56).

### 3.4. Net Benefit of Integrated ExoSpec Model

**Figure 4:**
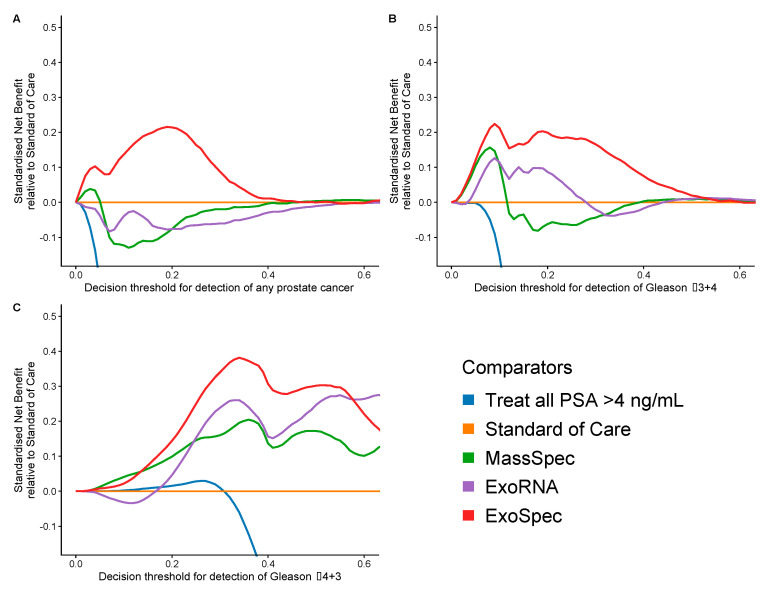
Estimation of standardised net benefit (sNB) of adopting each comparator model into clinical practice, displayed as decision curves, relative to standard of care. Accepted risk thresholds for the clinician before agreeing to biopsy are shown on the x-axis—decision threshold. For example, a clinician may accept up to a 25% perceived risk of significant cancer before recommending biopsy to a patient, equating to a decision threshold of 0.25. Each panel shows the relative sNB of a different biopsy outcome result when compared to standard of care: (**A**) detection of any prostate cancer, regardless of Gleason; (**B**) detection of Gleason ≥ 3 + 4; (**C**) detection of Gleason ≥ 4 + 3. The colour of each line represents mode model used: orange—biopsy of patients according to current standard of care, green—biopsy patients based on the MassSpec model, purple—biopsy patients based on the ExoRNA model, and red—biopsy patients based on the ExoSpec model. Data presented here were calculated from 1000 stratified bootstrap resamples of the available data to match the disease proportions reported from the control arm of the CAP study [37]. The mean sNB from these resamples was calculated and presented here.

**Figure 5:**
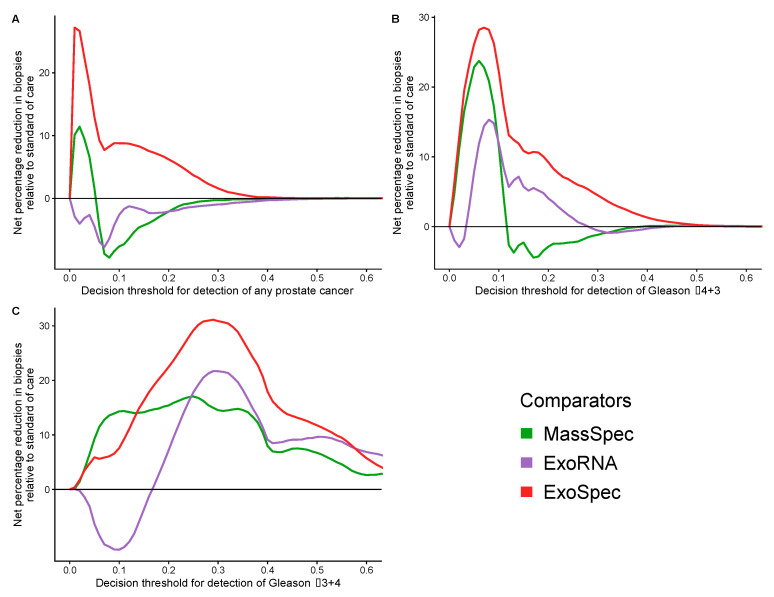
Potential reductions in unnecessary biopsies for each proposed model. Estimated from the net benefit of each model when compared to standard of care. Accepted risk thresholds for the interpreter before agreeing to biopsy are shown on the *x*-axis. Each panel details the estimated percentage reduction in biopsies for a differing biopsy outcome: (**A**) detection of any prostate cancer, regardless of Gleason; (**B**) detection of Gleason ≥ 3 + 4; (**C**) detection of Gleason ≥ 4 + 3. Green lines—biopsy patients based on the results of the MassSpec model, purple—the ExoRNA model, red—the ExoSpec model. The mean change in biopsies performed were calculated across 1000 stratified bootstrap resamples and presented here as a percentage.

Decision curve analysis (DCA) was used to examine the net benefit for each of the models in avoiding unnecessary biopsies, i.e., cancer negative or a Gs 3 + 3 biopsy result. DCA was performed on a population of patients suspected to harbour prostate cancer, using a PSA threshold of ≥4 ng/mL that can trigger further clinical investigations and biopsy [38]. The SoC model was taken as the baseline with which to compare DCA outputs from each of the three models: MassSpec, ExoRNA, and ExoSpec plus the result of biopsying all men with a PSA ≥ 4. The ExoSpec risk score consistently provided a net benefit across all decision thresholds and endpoints examined (ruling out all disease as well as high grade disease) and was the only model that was not predicted to be harmful at at least one threshold when compared to the SoC model (Figure 4). ExoSpec could result in a reduction in unnecessary biopsies by more than 30% for detecting clinically significant (Gs ≥ 3 + 4) disease across a range of reasonable accepted risk thresholds (0.1–0.3, Figure 5).

## 4. Discussion

Building upon our previous reports using single-omics features acquired by CE-MS proteomics [14] and EV-RNA data [11] from urine samples, in this manuscript we investigated if robust integration of these single-omics datasets via machine learning models can improve prediction of prostate cancer (PCa) found on biopsy. The integration of data from two very different technologies resulted in an increase in the predictive accuracy: from AUCs of 0.69, when using mass spectrometry peptide-metabolite data, and 0.75, when using extracellular vesicle RNA data, to 0.83 in the combined model. In comparison with other reported biomarkers, such as the 4K score test, PHI, PCA3, and SelectMDx, the results in this study have good performance (an AUC higher than 0.80 compared to 0.74–0.90) [39] and this justifies further investigations with larger cohorts. Detection of blood kallikreins such as in the 4K score and the prostate health index (PHI) have shown improvements over the PSA test in prostate cancer prediction. 4K and PHI have similar performances. The advantages of the PHI are that it is a cheap and simple blood test which measures PSA in three forms: total PSA, free PSA, and [-2]proPSA. However, the disadvantage of PHI is that [-2]proPSA levels have been reported to be unstable in blood which requires processing within 1 h for optimal results [40]. Secondly, these are PSA-based tests and blood levels of PSA can rise due to several causes besides cancer, such as benign prostatic hyperplasia [41], prostatic infection [42], or sexual intercourse [43]. Finally, PHI is used within the context of a PSA range of 4–10 ng/mL, and over 20% of clinically significant organ confined PCa occurs in men with a PSA less than 4 ng/mL [44,45]. The use of urine markers could sidestep these issues.

We have reported features as predictive biomarkers for significant PCa, including *ERG* exons 4–5, *PCA3*, *SERPINB5*, *SLC12A1*, *TMEM45B*, collagen alpha -1 (I) chains, and fibrinogen A (Table 2). These include NanoString detection of two well-established biomarkers for prostate cancer *PCA3* [46,47] and *TMPRSS2:ERG* [48]. *SERPINB5* is less well known; its transcript was decreased in PCa samples in this study, which would fit with its role in tumour inhibition, with loss or decreased expression of *SERPINB5* being reported in prostate cancer cells [49]. Several collagen and fibrinogen fragments have been previously reported as CE-MS biomarkers for discrimination of PCa patients from those without malignancy [50] and for detecting significant PCa [14]. In this study, all three fibrinogen peptides were identified at increased urinary abundance. Fibrinogen is reportedly overexpressed in urological cancers and is a key factor of tumour related inflammation and angiogenesis [51]. Levels of peptides from collagen types 1, 2, and 4 were significantly altered in urine samples from men with PCa. Interestingly MMP2, which was significantly increased in urine from PCa patients, can degrade collagen type 4 in basement membranes [52] forming a tentative link between some of the changes found in our studies.

NAD+ kinase (NADK) catalyses the phosphorylation of nicotinamide adenine dinucleotide (NAD+) to nicotinamide adenine dinucleotide phosphate (NADP+), which is subsequently reduced to NADPH [53]. As the demand for NADPH is particularly high in proliferating cancer cells, but also because it neutralizes the toxic high levels of reactive oxygen species (ROS) that are produced by increased metabolic activity, NADK has been implicated in several cancers and proposed as a target for therapeutic intervention [54]. In this study, NAD kinase was identified in increased urinary levels in PCa patients compared to ‘No Cancer’ samples. GLUD1, which is negatively allosterically regulated by NADP [55], was decreased. Some important features from the single-omics models did not add value to the combined ExoSpec model, which can be attributed in part to redundant information shared between the multiple datasets.

Higher accuracy in determining the risk of having aggressive prostate cancer before a diagnostic biopsy could reduce the number of men sent forward for unnecessary invasive biopsy. Our net benefit analyses demonstrated an added value over the standard of care assessment used for these patients and potentially could have reduced unnecessary biopsies by up to 30% dependent on accepted patient-clinician risk. Introduction of the test into the clinic should not be overly difficult. Urine is readily supplied by men in the clinic and can be frozen for transport to a specialized laboratory for analysis. Urine extracellular vesicles are much easier to purify than those in the blood stream and can simply be filtered out of the urine with a 100 kDa centrifugal filtration device. NanoString EV expression data and mass spectrometry data could be returned within 2 days. Feasibility and clinical applicability of the CE-MS based urinary peptidomics has been demonstrated among others in multicentric studies [56,57].

In this study, urine is utilized as a medium by which prostatic secretions are transported from the urethra to the outside world. We have examined our data relative to the strength of colour of urine samples and have found no link to the quality of the RNA expression data. We have subsequently introduced a urine preservative (Norgen, ON, Canada) which enables us to store urine samples at room temperature for up to 2 weeks without any loss of RNA quality; this will make transport for analysis simpler still [58].

Currently, the cost of the ExoSpec test per sample is high at approximately USD 920 (USD 120 for the EV expression data and ~USD 800 for the mass spec). This is mainly due to high instrument costs of mass spec. However, it is expected that a broader use of this approach will result in a lower cost. mpMRI and biopsy are also of considerable expense and so it is still possible that ExoSpec is cost effective considering the benefits in terms of reducing the number of biopsies and imaging. A full economic costings analysis should be performed before ExoSpec is implemented in the clinic.

There are some limitations to this study. In our cohort, prostate biopsy pathology was determined by TRUS biopsy, which will underestimate presence of significant cancer compared to template biopsy [5,59] when cancers are small [60]. A second limitation is that no MRI data was available for these men as samples were collected prior to MRI being widely phased in as a screening tool in the UK. Ahmed (2017) predicted that if TRUS biopsies were directed by MRI then up to 18% more clinically significant cancer would be detected [5]. However, while MRI can detect over 95% of significant disease (Gleason pattern ≥ 4), it does have a high false positive rate of ~50% [5]. We envisage that ExoSpec could perform well alongside MRI to reduce the number of negative biopsies taken as has been shown with other biomarkers such as PHI [61], but more research in this area is required [62]. Additionally, the development of ExoSpec was undertaken in a limited sample size for the number of predictors [63]; to compensate for this we implemented methods that are sufficiently robust to counter potential overfitting and bias, using strong internal validation methods in bootstrap resampling and out-of-bag predictions. These results are a starting point for validation of the predicted clinical benefits of the ExoSpec model in a prospective study.

## 5. Conclusions

The combination of biomarkers from multiple-omics sources improves our ability to detect significant prostate cancer (Gs ≥ 7) using urine samples. ExoSpec was able to accurately predict biopsy results and showed the potential for a large group of men to forgo an unnecessary invasive biopsy. If validated, ExoSpec has the potential to greatly improve the clinical care of men suspected to have prostate cancer.

## Figures and Tables

**Table 1:** Characteristics of the ExoSpec development cohort subdivided into ‘No Cancer’ (NC) and prostate cancer (PCa) patients—see Methods.

	‘No Cancer’ (NC)	PCa
**Collection Centre:** NNUH, *n* (%)	59 (100%)	133 (100%)
**Age (years):**		
minimum	45.0	53.0
median (IQR)	67.0 (59.5, 71.0)	70.0 (65.0, 76.0)
mean (sd)	66.2 ± 8.3	70.2 ± 7.8
maximum	82.0	91.0
**PSA (ng/mL):**		
minimum	0.3	4.10
median (IQR)	5.3 (2.3, 7.9)	10.40 (6.90, 16.60)
mean (sd)	6.4 ± 5.9	16.8 ± 17.4
maximum	30.3	95.9
**Prostate Size (DRE estimate):**		
Small, *n* (%)	16 (27%)	12 (9%)
Medium, *n* (%)	25 (42%)	67 (50%)
Large, *n* (%)	14 (24%)	38 (29%)
Unknown, *n* (%)	4 (7%)	16 (12%)
**Gleason Score:**		
0, *n* (%)	59 (100%)	0 (0%)
3 + 3, *n* (%)	0 (0%)	31 (23%)
3 + 4, *n* (%)	0 (0%)	48 (36%)
4 + 3, *n* (%)	0 (0%)	25 (19%)
≥4 + 4, *n* (%)	0 (0%)	29 (22%)
**Biopsy Outcome**		
No Biopsy, *n* (%)	23 (39%)	0 (0%)
Biopsy Negative, *n* (%)	36 (61%)	0 (0%)
Biopsy Positive, *n* (%)	0 (0%)	133 (100%)

The bold is used to aid visualisation of the separate variables.

**Table 2:** Features positively selected for each model.

	SoC	MassSpec	‘ExoRNA’	‘ExoSpec’	Difference (PCa vs. NC)
**Clinical Parameters**	Serum PSA	*-*	*-*	Serum PSA	**10.4** **×**
	Age	*-*	*-*	Age	**4.1** **×**
**Peptides**	-	**HIST1H1E** (KSPAKAKAVKPKAAKPKTAKPKAA)	-	**HIST1H1E** (KSPAKAKAVKPKAAKPKTAKPKAA)	**7.1** **×**
	-	**COL2A1** (RDGEPGTPGNpGPpGP)	-	**COL2A1** (RDGEPGTPGNpGPpGP)	**7.0** **×**
	-	**COL1A1** (GDDGEAGKpGRpGERGpPGP)	-	-	**6.4** **×**
	-	**FGA** (DEAGSEADHEGTHSTKRGHAKSRPV)	-	**FGA** (DEAGSEADHEGTHSTKRGHAKSRPV)	**5.6** **×**
	-	**COL4A4** (NEGLCACEpGpMGPPGPp)	-	-	**5.4** **×**
	-	**MMP2** (TAMSTVGGNSEGApCV)	-	-	**4.8** **×**
	-	-	-	**FGA** (ADHEGTHSTKRG)	**4.1** **×**
	-	-	-	**FGA** (SEADHEGTHSTKRG)	**3.2** **×**
	-	**NADK** (QTAPQEEAVTQEEVEALVCGHTQRWVPG)	-	-	**1.3** **×**
	-	**COL1A1** (ApGDRGEpGPPGp)	-	-	**0.7** **×**
	-	**COL1A1** (SpGPDGKTGPpGPA)	-	-	**0.6** **×**
	-	**COL4A3** (PGNEGLDGpRGDPGqPGpPGEqGP)	-	-	**0.6** **×**
	-	**COL4A5** (LPGFPGpEGPPGpRGQKGDDGIpGpPGPK)	-	-	**0.6** **×**
	-	**GLUD1** (AVGESDGSIWNPDGIDPK)	-	**GLUD1** (AVGESDGSIWNPDGIDPK)	**0.5** **×**
**EV-RNA** **probes**	-	-	*ERG* exons 4–5	*ERG* exons 4–5	**4.8** **×**
	-	-	*PCA3*	*PCA3*	**4.2** **×**
	-	-	*SLC12A1*	*SLC12A1*	**3.5** **×**
	-	-	*TMEM45B*	*TMEM45B*	**1.9** **×**
	-	-	*SERPINB5*	*-*	**0.8** **×**
	**-**	**-**	*SNORA20*	***-***	**0.8** **×**

Features were selected using cross-validated LASSO feature selection on four datasets: clinical variables only, mass spec, EV-RNA, and the combined dataset. Absolute mean differences between Prostate Cancer (PCa) and No Cancer (NC) samples; ‘×’ indicates linear fold-change values. Protein/gene names are provided with peptide sequences in brackets where appropriate. The *ERG* exon 4–5 probe will detect >95% of *TMPRSS2:ERG* splice-variant transcripts. Bold has been added for the peptides to add readability and clarity.

**Table 3:** Area under the receiver operator curve (AUC) values of all four trained models for detecting disease on an initial biopsy: (i) Any cancer, (ii) Gleason score (Gs) ≥ 3 + 4, (iii) Gs ≥ 4 + 3. SoC uses only clinically available information, MassSpec uses only peptide mass spectrometry data, ExoRNA uses only EV-RNA information, and ExoSpec is a multi-omics integrated model combining all data. Numbers within brackets are 95% confidence intervals for the AUC, calculated from 1000 stratified bootstrap resamples. Input variables for each model are detailed in Table 1.

Initial Biopsy Outcome	SoC	MassSpec	ExoRNA	ExoSpec
Any Cancer	0.78 (0.70–0.85)	0.76 (0.68–0.84)	0.84 (0.79–0.90)	0.91 (0.86–0.96)
Gs ≥ 3 + 4:	0.71 (0.64–0.78)	0.69 (0.61–0.76)	0.75 (0.68–0.82)	0.83 (0.77–0.88)
Gs ≥ 4 + 3:	0.76 (0.69–0.84)	0.70 (0.62–0.78)	0.67 (0.58–0.75)	0.82 (0.75–0.88)

## Data Availability

Raw normalised proteomics and NanoString data, along with the matched full clinical data, as well as the code and scripts that were applied in this manuscript and that are required to reproduce these analyses can be found at https://github.com/UEA-Cancer-Genetics-Lab/ExoSpec (accessed on 13 March 2022). Raw mass spectrometry data are available from the Zenedo repository platform at https://doi.org/10.5281/zenodo.6448114, accessed on 13 April 2022.

## Supplementary Materials

The following supporting information can be downloaded at: https://www.mdpi.com/article/10.3390/cancers14081995/s1, Table S1: List of predictive urinary peptides and NanoString probes that were employed for the construction of the predictive models.
